# Out-of-Pocket Payments, Catastrophic Health Expenditure and Poverty Among Households in Nigeria 2010

**DOI:** 10.15171/ijhpm.2018.19

**Published:** 2018-03-10

**Authors:** Bolaji Samson Aregbeshola, Samina Mohsin Khan

**Affiliations:** ^1^Department of Community Health & Primary Care, College of Medicine, University of Lagos, Lagos, Nigeria.; ^2^Department of Public Health Sciences, Karolinska Institutet, Stockholm, Sweden.

**Keywords:** Out-of-Pocket Payments, Catastrophic Health Expenditure, Poverty, Financial Risk Protection, Universal Health Coverag

## Abstract

**Background:** There is high reliance on out-of-pocket (OOP) health payments as a means of financing health system in Nigeria. OOP health payments can make households face catastrophe and become impoverished. The study aims to examine the financial burden of OOP health payments among households in Nigeria.

**Methods:** Secondary data from the Harmonized Nigeria Living Standard Survey (HNLSS) of 2009/2010 was utilized to assess the catastrophic and impoverishing effects of OOP health payments on households in Nigeria. Data analysis was carried out using ADePT 6.0 and STATA 12.

**Results:** We found that a total of 16.4% of households incurred catastrophic health payments at 10% threshold of total consumption expenditure while 13.7% of households incurred catastrophic health payments at 40% threshold of nonfood expenditure. Using the $1.25 a day poverty line, poverty headcount was 97.9% gross of health payments. OOP health payments led to a 0.8% rise in poverty headcount and this means that about 1.3 million Nigerians are being pushed below the poverty line. Better-off households were more likely to incur catastrophic health payments than poor households.

**Conclusion:** Our study shows the urgency with which policy makers need to increase public healthcare funding and provide social health protection plan against informal OOP health payments in order to provide financial risk protection which is currently absent among high percentage of households in Nigeria

## Background

### Out-of-Pocket Payments in Africa and Nigerian Healthcare System

There is high reliance on out-of-pocket (OOP) health payments as a means of financing health system in Nigeria. This has continued for many years in spite of a general consensus to move closer to universal health coverage (UHC) and sustain it when achieved.^1^ Advocates of user fees and direct payment for healthcare services which are forms of OOP health payments have rescinded on their argument and now agree that user fees should be removed due to its negative impact on poor people.^2^ According to the World Bank, OOP payments for health services determine whether a household would end up being poor or not.^3-5^ Communities from all over the world experience catastrophic health payments but people in low-income countries (LICs) are mostly affected.^6^ The World Health Organization (WHO) estimates that globally over 150 million people incur catastrophic health expenditure while over 100 million are pushed into poverty due to OOP health payments.^7^ Most low- and middle-income countries (LMICs) including Nigeria are battling the problem of poverty. Financial protection ensures that households do not face financial hardship and become impoverished as a result of seeking healthcare. In an attempt to address the lack of financial risk protection, the Federal Government of Nigeria in 2005 kick-started the National Health Insurance Scheme (NHIS) with the aim of providing access to good healthcare services and also ensuring the protection of households from the financial burden of OOP health payments. Over decade, evidence suggests that less than 5% of Nigerians mainly federal government workers are insured under this scheme.^8^ Also, less than 3% of the Nigerian population are under the private health insurance (PHI).^9^ Over 90% of Nigerians pay OOP for healthcare and this is supported by both WHO and World Bank statistics. Households who live below the poverty line often do not use healthcare services when the need arises.^2,10^ OOP health payments are capable of making households incur catastrophic health expenditure and this can exacerbate the level of poverty. OOP health payments are regarded as catastrophic when healthcare expenditure affects the ability of a household to purchase essential non-medical goods and services.^11^ In addition, a household can be said to be impoverished when health expenses displace finances that would have been used for both food and non-food items.^12^ However, OOP health payments, catastrophic health expenditure and poverty all have implications for household welfare and living standards. A number of studies in Africa have examined the financial burden of OOP health expenditure using different approaches. Results from these studies shows that OOP health payments have catastrophic and impoverishing effects on many households in Africa.^2,13-26^

### Overview of the Nigerian Health System

Nigeria is the most populous country in Africa with a population of about 186 million and a gross domestic product (GDP) of $405.1 billion in 2016.^27^ According to World Bank in 2016, Nigeria’s GDP per capita at purchasing power parity (PPP) was US$5867.^27^ The country is divided into six geo-political zones namely South East, South West, South South, North East, North Central and North West. Nigeria is also a political federation with 36 states and multi-ethnic groups. Nigerian health system has been evolving over the years^28^ but remains weak, inequitable and dysfunctional. The health system is a complex mixed system with private hospitals operating in a free market and public hospitals operating in a government controlled capacity. The private health sector is responsible for about 60% of healthcare service delivery while the public health sector account for 40%.^29^ The public health sector is on the verge of collapse due to inefficiency, poor infrastructure and poor resources. Although the local, state and federal governments are responsible for primary, secondary, and tertiary levels of care respectively, there is poor coordination of healthcare services among the three levels of health system in Nigeria due to duplication of responsibilities. Primary healthcare (PHC) system which is expected to be the foundation of the country’s health system has failed to provide basic healthcare services to the population due to problem of poor budgetary allocation, decaying and poor infrastructure, poor governance structure, poor service delivery and poor health worker performance.^30^ Furthermore, Nigeria has poor health indicators compared with the average for Africa region with life expectancy at 47.7 years, under five mortality rate at 108.8 per 1000 live births, maternal mortality ratio at 814 per 100 000 live births, infant mortality rate at 69 per 1000 live births and neonatal mortality rate at 34.3 per 1000 live births in 2017.^31^ Table 1 presents the statistics on health expenditure by type of healthcare financing agent as a percentage of total health expenditure.^32^ The high percentage of OOP health payments indicates that households contribute more to overall health expenditure than governments in Nigeria. The health financing and expenditure indices for Nigeria are also shown in Table 1.^33^ Alarmingly, OOP health expenditure as a percentage of private expenditure on health is 95%.^33^ The high level of OOP health spending as a major source of healthcare financing limits the ability of poor households to access and utilize basic healthcare services. In addition, Nigeria has the highest OOP health expenditure as a share of total health expenditure compared with African countries of which the highest OOP health expenditure is noted in Cameroon – 66%.^33^ This study aims to examine the extent to which households incur catastrophic OOP health expenditure and are impoverished as a result of OOP payments for healthcare services. The objectives of this study are to measure the incidence and distribution of catastrophic OOP health expenditure in Nigeria and examine the effect of OOP health payments on poverty in Nigeria.

**Table 1:** Financing Indicators in Nigeria

**Type of Financing Agents**	**Contribution to Overall Health Expenditure (%)**
NHIS	2
OOP payment	69
FMoH	7
SMoH	5
HMBs	4
LGA health departments	7
NGOs	0
Firms health department	1
Other federal agencies	5
**Health Financing and Expenditure Indices**	
Total health expenditure as a share of GDP	4
Public expenditure on health as a percentage of total health expenditure	24
Private expenditure on health as a percentage of total health expenditure	76
OOP expenditure as a percentage of total health expenditure	73
OOP health expenditure as a percentage of private expenditure on health	95

Abbreviations: NHIS, National Health Insurance Scheme; OOP, out-of-pocket; FMoH, Federal Ministry of Health; SMoH, State Ministries of Health; HMBs, Health Management Boards; LGA, local government area; NGOs, non-governmental organizations; GDP, gross domestic product. Source: Uzochukwu et al 2015^32^ and WHO.^33^

### Contribution of the Study

There is limited evidence on the catastrophic and impoverishing effects of OOP health payments in Nigeria using a nationally representative household survey data.^34-36^ Therefore, there is a need to provide evidence on the catastrophic and impoverishing effects of OOP health payments in Nigeria in order to inform governments and policy makers on the necessity of designing programs and policies that would provide financial risk protection to populations as a target of Sustainable Development Goals (SDGs). This study attempts to monitor trends of financial risk protection and evaluate the impact of health financing reform such as the NHIS since its operationalization in 2005. Previous studies on the catastrophic and impoverishing effects of OOP health expenditure in Nigeria were conducted at the state level with the exception of one study which was conducted using the 1999 General Household Survey of the Federal Office of Statistics. However, our study differs from these previous studies. The present study used the 2009/2010 nationally representative household living standard survey data and followed the guidelines for assessing health equity by the World Bank.^37^ This research also differs from studies conducted on the catastrophic and impoverishing effects of OOP health expenditure elsewhere by comparing findings among African countries. Our study contributes to the literature in Africa and to better understanding of the impact of OOP health payments on catastrophic health expenditure and impoverishment in Nigeria.

### Organisation of the Research Paper

The rest of this research article is organized as follows: Section 2 describes the HNLSS 2009/2010 data, data analysis and outlines the methodologies to measure catastrophic OOP health payments and the impoverishing effects of OOP health payments. Section 3 presents the empirical results of the study. Section 4 discusses empirical results in comparison with findings from other African countries. Section 5 concludes with suggestions for policy-makers in Nigeria and international audience.

## Methods

Secondary data from the Harmonised Nigeria Living Standard Survey (HNLSS) 2009/2010 was used for the study. HNLSS is a nationally representative cross-sectional study conducted by the National Bureau of Statistics (NBS) with funding by United Kingdom Department for International Development (UK DFID) and the World Bank as a follow up to the Nigeria Living Standard Survey (NLSS) of 2003/2004. The HNLSS 2009/2010 has an enlarged scope that includes health, household income, consumption and expenditure, demography, education and skill/training compared with previous surveys. HNLSS is a combination of NLSS household questionnaire and Core Welfare Indicator Questionnaire (CWIQ) jointly developed by NBS and the World Bank. Thirty-six states of the federation and the federal capital territory (FCT), Abuja was covered in the survey. The first part of the survey using the welfare approach was conducted among 77 400 households while the second part of the survey using the consumption approach covered 50 households in each local government area (LGA). In total, 38 700 households were interviewed. Sampling frame for the survey used enumeration areas (EAs) specified by the National Population Commission (NPC). Two stage sample design was employed. The first stage involved the selection of EAs while the second stage involved the selection of households. Data was collected through interviews conducted by NBS enumerators with household members on a quarterly basis from November 2009 to October 2010.

### Data Analysis

Data analysis was carried out using ADePT version 6.0 and STATA version 12 software.

### Measuring Catastrophic Effect of Out-of-Pocket Health Payments

There is no consensus in the existing literature and among health economists on the threshold proportion of household expenditure.^37^ However, there is an agreement that catastrophic health expenditure are medical spending or OOP health expenditure that exceed a defined threshold of household’s total consumption or non-food consumption expenditure annually.^6,38-40^ Two methods are generally used in measuring catastrophic health payments. They include estimating catastrophic health expenditures with health expenditures as a share of total expenditures and non-food expenditures^37,40^ and as a share of capacity to pay.^41^ Some scholars have argued that these two methods do not consider household’s external resources. Regardless of the arbitrary nature of thresholds used, majority of previous studies on defining catastrophic payments used methods proposed by Wagstaff and van Doorslaer,^40^ O’Donnell et al,^37^ and Xu.^41^

This study employed the approach used by Wagstaff and van Doorslaer^40^ to measure catastrophic payments for healthcare as in previous studies.^2,35,42-46^ The method for estimating catastrophic effect of OOP health payment is well known and described in details elsewhere.^16^

### Measuring Impoverishing Effect of Out-of-Pocket Health Payments

OOP health payments should not push households that are above the poverty line into poverty and those below the poverty line deeper into poverty. The impoverishing effect of OOP health payments examines the extent of poverty due to OOP health spending incurred by households.^40^ The methodology developed by Wagstaff van Doorslaer^40^ was used to estimate the impoverishing effect of OOP health payments. This method adjusted poverty measures based on household expenditure net of OOP payments on healthcare. Poverty headcount, poverty gap, normalized poverty gap and normalized mean poverty gap were used to measure poverty. In calculating these measures of poverty, the international poverty line of $1.2 and $2.0 per day developed by World Bank with PPP values of 2005 was used. These poverty lines were then deflated using the consumer price index (CPI) for 2009 using data from the World Development Index (WDI) database also developed by the World Bank. The extent to which OOP health payments pushed households below the poverty line was assessed thereafter. The method for estimating impoverishing effect of OOP health payment is well known and described in details elsewhere.^16^

## Results

### Descriptive Statistics

Table 2 presents the population characteristics of households and individuals involved in the study. The study sample is representative of the general Nigerian population.

**Table 2:** Demographic and Socio-Economic Profile of the Study Population

**Household and Individual Characteristics** **(N = 305 000)**	**Percent (No.)**
Age	
0-5	13.8 (42 015)
6-14	24.8 (75 593)
15-24	18.4 (56 239)
25-54	33.3 (101 588)
55-64	5.1 (15 409)
65 and above	4.6 (14 156)
Education of household head	
None	46.6 (142 043)
Nursery	0.1 (246)
Primary	31.6 (96 381)
Secondary	16.7 (50 873)
Post-secondary	5.1 (15 457)
Gender of household head	
Male	50.9 (155 206)
Female	49.1 (149 794)
Household size	
Less than 5 members	22.5 (68 724)
More than 5 members	77.5 (236 276)
Location	
Urban	25.9 (79 116)
Rural	74.1 (225 884)
Geo-political zone	
North Central	16.9 (51 693)
North East	12.5 (38 263)
North West	27.7 (84 502)
South East	12.3 (37 663)
South South	15.0 (45 755)
South West	15.5 (47 124)
Socio-economic status	
Poorest	0.1 (260)
Poorer	64.1 (195 651)
Middle	35.7 (108 786)
Richer	0.1 (240)
Richest	0 (63)
Work status of household head	
Employed	62.8 (191 628)
Unemployed	37.2 (113 372)
Health insurance status	
Lack health insurance	77.9 (237 728)
Have health insurance	22.1 (67 332)
Type of health facility visited	
Public health facility	95.3 (290 531)
Private health facility	4.7 (14 469)
Type of illness suffered	
Non-chronic illness	99.2 (302 408)
Chronic illness	0.8 (2592)

### Catastrophic Effect of Out-of-Pocket Health Payments

**Figure 1:**
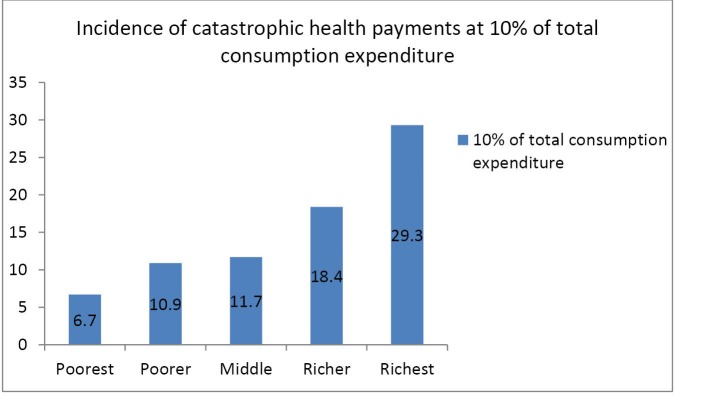
Incidence of Catastrophic Out-of-Pocket (OOP) Health Payments by Quintile Using 10% of Total Consumption Expenditure.

**Figure 2:**
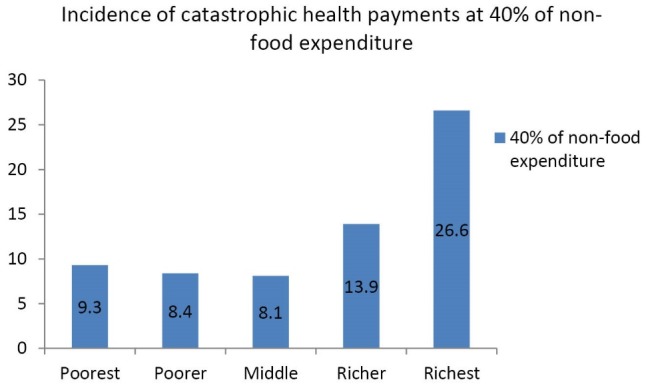
Incidence of Catastrophic Out-of-Pocket (OOP) Health Payments by Quintile Using 40% of Total Consumption Expenditure.

Table 3 present results of the incidence and distribution of catastrophic health payment at thresholds ranging between 5% to 40% for both total household expenditure and household non-food expenditure. At the threshold of 5% of total consumption expenditure catastrophic head count ratio was at 18.2%. This decreased to 16.4% at 10% threshold of total consumption expenditure. Only 13.6% of households incurred OOP health payments on healthcare in excess of 25% of total consumption expenditure while 12.3% of households incurred catastrophic health payments at 40% threshold of total consumption expenditure. Furthermore, at 5% threshold of non-food expenditure, 20.5% of households incurred catastrophic health payments. This decreased to 18.6% at 10% threshold of non-food expenditure. Only 15.5% of households incurred OOP health payments in excess of 25% of non-food expenditure while 13.7% of households incurred catastrophic health payments at 40% threshold of non-food expenditure. The positive concentration index shows that the intensity of catastrophic health expenditure affects the better-off households more than the poor (Table 3). Figure 1 shows the proportion of households that incurred catastrophic health payments at 10% threshold of total consumption expenditure while Figure 2 shows the proportion of households that incurred catastrophic health payments at 40% threshold of non-food expenditure.

**Table 3:** Incidence and Distribution of Catastrophic OOP Health Payments

	**5%**	**10%**	**15%**	**25%**	**30%**	**40%**
**Threshold Budget Share (%) of Total Expenditure**						
Headcount (H)	18.2	16.4	15.3	13.6	13.2	12.3
Concentration index, C_E	0.202	0.221	0.233	0.251	0.259	0.269
Concentration index, C_O	0.261	0.266	0.270	0.276	0.279	0.282
** Threshold Budget Share (%) of Non-food Expenditure**						
Headcount (H)	20.5	18.6	17.5	15.5	14.8	13.7
Concentration index, C_E	0.244	0.266	0.284	0.310	0.315	0.339
Concentration index, C_O	0.841	0.841	0.841	0.842	0.842	0.842

Abbreviation: OOP, out-of-pocket. Source: Author’s estimates using ADePT and data from HNLSS 2009/2010.

### Impoverishing Effect of Out-of-Pocket Health Payments

Table 4 presents results for the impoverishing effect of OOP health payments. Using the World Bank $1.2 a day poverty line, as many as 97.9% of households is estimated to be in poverty based on total consumption expenditure. This increases to 98.7% when OOP health payments are netted out of total consumption expenditure. Only 0.8% of households are not living in poverty but would be pushed into poverty if OOP health spending were netted from total consumption expenditure. OOP health payments led to a 0.8% rise in poverty headcount ratio. This represents 1 268 800 Nigerians being pushed below the poverty line due to OOP health payments. There is also a relative rise of 0.8% in the estimate of extreme poverty. Poverty gap rises from 2492.2 Naira (US$ 16.40) to 2539.8 Naira (US$16.7). Normalised poverty gap increased from 92.8% to 94.6% while the normalised mean positive poverty gap increased from 94.8% to 95.9%. Therefore, poverty gap rises as more households are pushed into poverty. Using the World Bank poverty line of $2.0 per day, the observations and patterns of result were similar. There was an increase in the number of households impoverished as a result of OOP health payments. The relative rise in poverty was 0.4%.

**Table 4:** Impoverishment Impact of OOP Health Payments

	**Gross OOP payments (A)**	**Net OOP payments (B)**	**Absolute (B) – (A) = (C)**	**Relative (C)/(A)x100**
Poverty line = PL1 ($1.25 per day)				
Poverty headcount (%)	97.9	98.7	0.8	0.8
Poverty gap (Naira)	2492.2	2539.8	47.6	1.9
Normalized poverty gap (%)	92.8	94.6	1.8	1.9
Normalized mean positive poverty gap (%)	94.8	95.9	1.1	1.2
Poverty line = PL2 ($2.00 per day)				
Poverty headcount (%)	98.7	99.1	0.4	0.4
Poverty gap (Naira)	4077.3	4133.9	56.6	1.4
Normalized poverty gap (%)	94.9	96.2	1.3	1.4
Normalized mean positive poverty gap (%)	96.1	97.0	0.9	0.9

Abbreviation: OOP, out-of-pocket. Note: Between 2009 and 2010 when the data was collected, the interbank exchange rate was 1US$ = 152 Naira.

## Discussion

This study used the 2009/2010 HNLSS dataset that was collected five years after the operationalization of NHIS. Findings from our study indicate that the Nigerian health system does not protect majority of the population from the effects of OOP health payments.

### Catastrophic Effect of Out-of-Pocket Health Payments

Households in Nigeria incurred catastrophic OOP health payments. At 10% threshold of total consumption expenditure, Nigeria had higher catastrophic effect of OOP health payments compared with African countries such as Ghana,^2^ Kenya,^19-21^ Mongolia,^15,16^ Senegal,^23^ Zambia,^18^ and Swaziland^26^ but is better off than Uganda^24^ and Egypt.^17^ Details are given in Table 5. At 40% threshold of non-food expenditure, Nigeria had higher catastrophic effect of OOP health payments compared with Egypt,^17^ Kenya,^19-21^ Mongolia,^15,16^ Malawi,^47^ Ghana,^2^ Burkina Faso,^25^ Zambia,^18^ and Swaziland^26^ but is better off than Tanzania.^22^ Details are also given in Table 5.

**Table 5:** Catastrophic and Impoverishing Effects of OOP Health Payments in African Countries

**Country**	**Catastrophic Effect of OOP Health Payments**		**Impoverishing Effect of OOP Health Payments**	
	**10% of Total Health Expenditure**	**40% of Non-Food Expenditure**	**Absolute Poverty**	**Relative Poverty**
Tanzania (2014)	-	18%	-	-
Burkina Faso (2006)	-	10.8%	-	-
Ghana (2010)	5.2%*	2.4%	1.6	9.4%
Kenya (2017)	-	6.6%	1.6	2.4%
Kenya (2016)	14.3%	9.8%	3.1	6.3%
Kenya (2012)	15.5%	11.4%	2.7	5%
Uganda (2015)	22.8%	-	4.1	18.1%
Malawi (2017)	-	0.7%	0.9	1.8%
Mongolia (2016)	5.5%	1.1%	1.6	12.0%
Mongolia (2012)	10%	3.3%	2.5	7.0%
Egypt (2015)	22.4%	7.1%	0.4	66.6%
Senegal (2015)	6.3%	-	-	-
Zambia (2016)	9.3%	11.2%	-	-
Swaziland (2015)	9.6%	2.7%	1.6	7.7%

Abbreviation: OOP, out-of-pocket. Source: Brinda et al 2014^22^; Su et al 2006^25^; Akazili 2010^2^; Barasa et al 2017^21^; Kimani et al 2016^20^; Chuma and Maina^19^; Kwesiga et al 2015^24^; Mchenga 2017^47^; Dorjdagva et al 2016^16^; Bredenkamp et al 2012^15^; Rashad and Sharaf 2015^17^; Sene and Cisse 2015^23^; Masiye et al 2016^18^; Ngcamphalala 2015.^26^ * The values are rounded to 1 decimal.

In this study, better-off households are more likely to spend a large fraction of total household resources on healthcare. The empirical finding that the better-off households are more likely to incur catastrophic health payments than poor households in Nigeria is supported by similar studies conducted in Mongolia,^15,16^ Egypt,^17^ Nigeria,^35^ Asia,^46^ and Cambodia.^48^ A possible explanation is that poor households may seek low quality care, avoid seeking healthcare at all or resort to self-medication due to inability to pay for healthcare services.^49^ Also, better-off households may have been by-passing low quality public PHC facilities for private healthcare facilities that are expensive. Free healthcare services and exemption mechanisms that are occasionally provided by governments at the national and sub-national levels to vulnerable populations could also explain why catastrophic OOP health payments are disproportionally concentrated among the better-off in Nigeria.^50^

### Impoverishing Effect of Out-of-Pocket Health Payments

Some households are pushed into poverty due to OOP health payments. Using the relative poverty ratio, the proportion of households that are impoverished due to OOP health payments was lower in Nigeria compared with African countries like Mongolia,^15,16^ Ghana,^2^ Uganda,^24^ Malawi,^47^ Kenya,^19-21^ Egypt,^17^ and Swaziland.^26^ Details are given in Table 5.

The high percentage of catastrophic health expenditure and poverty in Nigeria shows that health financing reform such as the NHIS which became fully operational in 2005 is not achieving its aim of reducing the financial burden of OOP health payment among households. Although the study used 2010 data to inform policy-makers, there is lack of change in the health insurance system in the last seven years (2010-2017) as evidenced by the low coverage of health insurance in Nigeria. Over 10 years after the operationalization of NHIS, less than 10% of the Nigerian population are insured in both the formal and informal sectors. NHIS has not expanded to capture the over 90% who pay OOP for healthcare. PHI which is a healthcare financing strategy with the potential to address the issue of insufficient government health spending has so far contributed little. According to WHO in 2016, PHI expenditure as a share of total health expenditure was 2%.^33^ Furthermore, PHI in Nigeria is voluntary rather than mandatory—this is responsible for low coverage among households. The high incidence of catastrophic health payments among better-off households as found in this study shows that OOP health payments are progressive and remain an inequitable form of financing health system. Pre-payment mechanisms have been advocated as the best option for reducing financial catastrophe.^12,51,52^ Evidence from countries such as Thailand, Mexico, Rwanda, Sri Lanka, and Malaysia show that mandatory pre-payment mechanisms increase financial risk protection thereby reducing the burden of OOP health expenditure on both the poor and the better-off groups.^5,53,54^ However, pre-payment mechanism such as tax and health insurance have not been able to finance the health needs of the poor and vulnerable populations who are mostly in the informal sector of the economy. Therefore, NHIS need to be expanded to cover majority of households that are in the informal sector.

Since the operationalization of NHIS in 2005, the provision of health insurance remains optional for states with the exception of states such as Cross Rivers and Bauchi States that have provided health insurance coverage to residents while some other states have indicated their interest.^32^ This low buy-in by most states across Nigeria necessitate state health financing reforms to tackle the problem of high levels of OOP health spending in Nigeria. Although anecdotal evidence suggests that the pilot performance-based financing (PBF) scheme in Ondo, Nassarawa, and Adamawa States have led to improved quality of care, improved health worker motivation and increased utilization of maternal and child healthcare services. There is however a significant variation between the three states.^55^ Taking ownership of such health financing strategy by state governments and aligning them with health system reforms will help in addressing the problem of poor quality of care and low utilization of healthcare services by poor households.

It is clear from our study that poor households who mostly reside in rural areas are excluded from the Nigerian health system due to unaffordable healthcare cost and the inability to pay premiums under the NHIS program for the informal sector, hence, the need to provide a social health protection plan targeted at these households. This can be achieved by establishing a government-funded social health protection scheme through a general tax financing system for the poor and vulnerable populations.

## Limitations of the Study

Our study has some limitations but this does not invalidate our work because all the steps towards measuring financial risk protection were strictly followed. The study used nationally representative cross-sectional survey rather than longitudinal approach due to the challenges that accompany longitudinal household surveys. Another limitation of the study is recall bias. Recall bias among households could affect the accuracy of data collected. Despite these limitations, the findings from this study provide important evidence on the financial burden of OOP health payments on households in Nigeria and would inform policy-makers on the need to stop the high reliance of OOP health payments towards achieving financial risk protection as a goal of UHC.

## Conclusion

Our study shows that OOP health payments led to catastrophic health expenditure and exacerbated poverty. The NHIS has not provided financial risk protection to the population. Households, however, have high financial burden due to OOP health payments. This implies that they have limited access to quality healthcare services and face financial hardship as a result of seeking healthcare. There is a need for political actors and policy-makers to design health system financing policies that will provide financial risk protection to households. Evidence suggests that increased allocation of public funds to the health sector leads to a decrease in OOP health expenditure as well as catastrophic OOP health spending.^33^ Thus, the insufficient public health financing over the years is a major driver for high levels of OOP health spending in Nigeria. Our study shows the urgency with which policy-makers need to increase public healthcare funding and provide social health protection plan against informal OOP health payments in order to provide financial risk protection which is currently absent among the high percentage of households in Nigeria. There is a need to pay proper attention to the health of Nigerian population and address issues such as OOP health payments that increase the level of poverty which is an indicator of poor economic growth.

## Policy Implications

Results from our study have policy implications for policy-makers in Nigeria and similar countries. First, governments have to significantly increase public spending on health. Domestic financial resources are key to moving closer to UHC and should be increased on a long-term basis. The Abuja declaration of 2001 in which African heads of state pledged to set a target of earmarking at least 15% of their annual budget to improve the health sector needs to be fully implemented by governments at the national and sub-national levels. Second, PHC system needs to be strengthened and PHC facilities made functional with the provision of comprehensive benefit package for the poor and vulnerable populations in order to improve access to healthcare services and health outcomes. A pro-poor policy reform with improved quality of care, availability of essential medicines and equitable distribution of health workers will improve coverage and utilization of healthcare services for the poor and most vulnerable households. Lastly, results from this study provide a guide for future health financing reforms and a baseline for further research on financial risk protection in Nigeria.

## Ethical issues

Secondary data were used for this study. In obtaining the micro data, a request was made on the National Bureau of Statistics website and approval was granted to download the data, hence, there were no ethical issues of concern. These data are public and freely available to anyone from National Bureau of Statistics on request. The website for NBS is http://nigerianstat.gov.ng/nada/index.php/catalog/.

## Competing interests

Authors declare that they have no competing interests.

## Authors’ contributions

BSA: Conception and design; acquisition of data; analysis and interpretation of data; and drafting of the manuscript. SMK: Analysis and interpretation of data; and drafting of the manuscript. Both authors reviewed the manuscript for important intellectual content and approved the final draft for publication.

## Authors’ affiliations

^1^Department of Community Health & Primary Care, College of Medicine, University of Lagos, Lagos, Nigeria. ^2^Department of Public Health Sciences, Karolinska Institutet, Stockholm, Sweden.

## Key messages

**Implications for policy makers**

- Findings from our study provide important insight on the impact of health financing reform such as the National Health Insurance Scheme (NHIS) and show that policy-makers need to reduce the reliance on out-of-pocket (OOP) health payments and provide social health protection plan against informal OOP health payments for households.
- Results also show that catastrophic OOP health payments are disproportionally concentrated among the better-off households in Nigeria possibly due to poor utilization of healthcare service by poor households, free healthcare services and exemption mechanisms; and the by-pass of low quality public primary healthcare (PHC) facilities by better-off households; hence, policy-makers need to design policies that will ensure that resources for healthcare are equitably distributed and benefit both the poor and better-off households.
- Governments have to significantly increase public spending on health. Domestic financial resources are key to moving closer to universal health coverage (UHC) and should be increased on a long-term basis. The Abuja declaration of 2001 in which African heads of state pledged to set a target of earmarking at least 15% of their annual budget to improve the health sector needs to be fully implemented by governments at the national and sub-national levels.
- PHC system needs to be strengthened and PHC facilities made functional with the provision of comprehensive benefit package for the poor and vulnerable populations in order to improve access to healthcare services and health outcomes. A pro-poor policy reform with improved quality of care, availability of essential medicines and equitable distribution of health workers will improve coverage and utilization of healthcare services for the poor and most vulnerable households.
- The lack of financial risk protection in Nigeria’s health system is a major challenge that policy-makers have to urgently address towards achieving UHC as a target of Sustainable Development Goals (SDGs). Results from this study provide a guide for future health financing reforms and a baseline for further research on financial risk protection in Nigeria.

**Implications for the public**

Out-of-pocket (OOP) health payments have been found to be a progressive and inequitable form of financing health system with catastrophic and impoverishing effects on household living standards. The high reliance on OOP health expenditure as reported in this study impact negatively on access to quality healthcare and household’s financial risk protection. Results from our study suggest the need for the general public and media outlets to embark on the advocacy to reduce OOP health payments and demand for health system reforms from governments at the national and sub-national levels.
